# High phenanthrene degrading efficiency by different microbial compositions construction

**DOI:** 10.3389/fmicb.2024.1439216

**Published:** 2024-08-30

**Authors:** Guoyan Zhou, Hongtao Qiao, Yandong Liu, Xiongsheng Yu, Xiang Niu

**Affiliations:** ^1^Department of Chemistry, Xinzhou Normal University, Xinzhou, Shanxi, China; ^2^Department of Biology, Xinzhou Normal University, Xinzhou, Shanxi, China; ^3^Shaoxing Academy of Agricultural Sciences, Shaoxing, China

**Keywords:** degrading bacteria, microbial communities, microbial domestication, phenanthrene degradation, polycyclic aromatic hydrocarbons

## Abstract

Microbial remediation has become the most promising technical means for the remediation of polycyclic aromatic hydrocarbons (PAHs) non-point source contaminated soil due to its low cost of treatment, complete degradation of pollutants, and *in-situ* remediation. In this study, in order to demonstrate the phenanthrene degrading microbial diversity, phenanthrene was chosen as the representative of PAHs and strains capable of degrading phenanthrene were isolated and screened from the sedimentation sludge and the bottom sludge of oil tank trucks, and high throughput sequencing was used to check the dominant strains with a good degrading effect on phenanthrene. Results showed even more than 50% of phenanthrene was degraded in all samples, the composition of PAH-degrading bacteria was diverse, and different environments constructed different functional microbial groups, which resulted in the microbial adapting to the diversity of the environment. Finally, a series of bacterial species with phenanthrene-degrading functions such as *Achromobacter, Pseudomonas, Pseudochelatococcus, Bosea* was enriched after nine transferring process. Overall, our study offers value information for the enrichment of functional degrading microbes of phenanthrene or other pollutants that more concern should be paid in not only the degradation rate, but also the diversity variation of microbial community composition.

## Introduction

Polycyclic aromatic hydrocarbons (PAHs) are a class of hydrocarbons with two or more benzene rings, and their molecules are mostly arranged in linear, angular or cluster arrangements (Dearden, 2003; Wang et al., 2013). PAHs have high melting and boiling point and strong hydrophobicity. With the increase of the number of benzene rings of PAHs, their water solubility gradually decreases, the difficulty of degradation in the environment increases, and the toxicity to organisms also increases rapidly. In addition, PAHs also have characteristics such as lipophilicity, photosensitivity, and high melting and boiling points (Kim et al., 2013; Abdel-Shafy and Mansour, 2016). PAHs entering the environment can be migrated and transformed through complex physical, chemical or biological pathways, such as volatilization, leaching, runoff, adsorption, photolysis, chemical oxidation and biological processes (Thion et al., 2013; Li et al., 2024). In PAHs-contaminated soils, bacteria are the main bearer of PAHs degradation (Bamforth and Singleton, 2005; Haritash and Kaushik, 2009). Microbial degradation has become the most promising technical means for PAHs removal in the soil polluted by organic pollution because of its low treatment cost, and from the current global research, PAHs bioremediation is a complex system engineering with good application prospects (Alrumman et al., 2016). Thus, it is essential to dig out the high PAHs degradation efficiency microbes.

As one of the widely used PAHs, phenanthrene (PHE) contains three-ring polycyclic aromatic hydrocarbon and was commonly found in various environments e.g. soils and rivers. Microbial diversity is closely related to phenanthrene remediation efficiency (Jain et al., 2005; Zhang et al., 2023a,b). Many typical degrading bacteria that can degrade PHE, including *Pseudomonas* and *Pseudarthrobacter* (Naloka et al., 2024), *Bacillus subtilis* (Lyu et al., 2023), *Achromobacter* (Hou et al., 2018), *Sphingobium* (Shin et al., 2011; Zhang et al., 2023b) were found in previous studies. Motterãn et al. (2024) showed *Bacillus* and *Alcaligenes* were enriched and isolated as potential degraders for phenanthrene from an industrial wastewater treatment plant. Zavala-Meneses et al. (2024) investigated a newly isolated strain *Pseudomonas veronii* SM-20 with phenanthrene as a sole carbon and energy source. They showed that the stain SM-20 could degrade phenanthrene through oxidation and ring-cleavage processes. In addition, research results have found that the effect of microflora on PHE degradation was better than that of the single strain, which was mainly due to the cooperation between microbial communities (Motterãn et al., 2024). A previous study used stable isotope probing (SIP) to assess and identify active phenanthrene degraders and showed fungal groups of *Crypotococcus, Cladosporium* and *Tremellales* and bacterial groups of *Alcanivorax, Marinobacter* and *Enterococcus* were predicted microbial community that could actually degrade phenanthrene (Schwarz et al., 2018).

In recent years, increasing attention has been paid to the functional redundancy and stability of microbial communities (Liu et al., 2021; Su et al., 2022). And the theory of functional redundancy is often used in ecological microbial models (Galand et al., 2018). Because broad taxa may have similar metabolic functions, the composition of microbial communities does not always alter ecosystem processes. Functional redundancy is therefore considered to be a ubiquitous phenomenon in microbial communities (Louca et al., 2018). A series of studies conducted in a variety of settings from nature to the human body have shown that microbial communities exhibited a high degree of “functional redundancy” across multiple functions (Turnbaugh et al., 2009; Burke et al., 2011; Louca et al., 2016). The loss of a small number of bacteria does not usually affect their function because multiple species can perform the same function. This functional redundancy may explain why the number of microbial species (alpha diversity) is independent of environmental function (Yu and Whalen, 2020). The stabilization mechanisms of biodiversity, including traditional stability theories, have been the focus of many studies on the long-term stability of ecosystems (Loreau and De Mazancourt, 2013; Isbell et al., 2015). According to theoretical and experimental studies, functional composition significantly affects microbial community stability under environmental change. Microbial communities rely on complex interactions among internal members to maintain stability and resilience to environmental perturbations (Pillar et al., 2013; Polley et al., 2013; Craven et al., 2018; de Bello et al., 2021). And due to the existence of bacterial functional redundancy, the increase in bacterial community diversity index may have a positive or negative impact on the remediation effect. Similarly, its impact on soil microbial characteristics also depends on the degree of bacterial functional redundancy (Girvan et al., 2005). But how the microbial communities changed and whether or not microbial functional redundancy existed during microbial domestication process is still unclear.

We used contaminated tank truck bottom sludge and precipitated sludge as samples, and isolated and screened local degradable bacteria with polycyclic aromatic hydrocarbon of phenanthrene as the only carbon source required for microbial growth and metabolism. These two soils were selected due to their high possibility to degrade PHE by indigenous microbial communities. The microbial degradation efficiency of phenanthrene was explored, and the dominant bacterial community with a high degradation effect on phenanthrene was detected. We hypothesized that the composition of PHE-degrading bacteria is diverse, which is mainly due to the result of different communities created by different environments, and the production of degradation functions is a community co-action rather than an individual.

## Materials and methods

### Chemicals

Phenanthrene (purity 95%) and pyrene (purity 99.9%) are purchased from Shanghai Aladdin Biochemical Technology Co., Ltd. Acetone, n-hexane, dichloromethane, all chromatographic pure. Other required drugs are pure analysis, all purchased from Sinopharm Chemical Reagent Shanghai Co., Ltd.

### Culture medium

The composition of liquid inorganic salt medium: 0.50 g L^−1^ NaNO_3_, 1.0 g L^−1^ KH_2_PO_4_, 0.20 g L^−1^ MgSO_4_, 0.020 g L^−1^ CaCl_2_, 1.0 g L^−1^ NaHPO_4_, 0.50 g L^−1^ (NH_4_)_2_ SO_4_, pH = 7.0, 121°C, sterilization for 20 min. 50 mg L^−1^ phenanthrene-acetone solution: weighing 0.005 g phenanthrene dissolved in acetone, set the volume in a 100 mL volumetric flask, and stored it in the −20°C of the refrigerator. 50 mg L^−1^ pyrene-acetone solution: weighing 0.0050 g of pyrene dissolved in acetone and stored in the −20°C of the refrigerator.

### Microbial enrichment and domestication

In order to study the variation t of PHE degrading microbial communities in different soil samples under different conditions, two kinds of soil samples of contaminated sludge (W), and tank truck sludge (D) were used in this study. Four hundred microliter of 50 mg L^−1^ PHE and or pyrene was added into glass tubes and d the solvent was volatilized before adding 20 ml inorganic salt medium. 2.00 g soil was then added into the medium and the tubes was sealed with sealing membrane and wrapped with tin foil. All operations were performed in the ultra-clean bench. Three treatments were set up with contaminated sludge containing PHE (EW), contaminated sludge containing PHE and pyrene (ERW) and tank truck sludge containing PHE and pyrene (ERD). Besides, control treatment without soil addition was also conducted in the test. For each treatment, independent biodegradation assays were performed in triplicate. All cultures were incubated in a thermostatic oscillator (30°C, 180 rpm) for 7 days, and were ventilated in an ultra-clean platform every two days for two minutes each time. After 7 days incubation, 5 ml of the incubated microbial supernatant was transferred to a new glass tube with 20 ml salt medium containing 1 mg L^−1^ PHE. The above domestication process was repeated nine times, and the concentration of PHE and microbial communities were detected each time.

### Determination of phenanthrene degradation rate and microbial diversity

The concentration of phenanthrene was detected following previous study of Xu et al. (2021). Briefly, an equal volume of dichloromethane was added to the sample and then vortexed for 30 s, sonicated for 30 min, and then centrifuged at 2,000 rpm for 5 min. After obtaining the above extract, anhydrous sodium sulfate was used to remove water before it was detected. A gas chromatograph (GC) (Agilent 7890 A, Agilent, Santa Clara, CA, USA) and HP-5 MS capillary column (30 m,0.32 mm diameter, 0.25 mm) (J & W Scientific Inc., CA, USA) were used. The operating conditions of the instrument are as follows: the initial temperature was set to 70°C for 2 min, increased to 300°C at 40°C min^−1^, and then kept constant for 3 min.

The collected samples were used to extract DNA and sequencing. Soil DNA Kit was used to extract the microbial communities (Dineen et al., 2010). The concentration and purity of DNA were detected using a spectrophotometer of nanosized DNA. Bacterial PCR amplification using primers pairs 338F and 806R, PCR using Quant-iT PicoGreen dsDNA Assay Kit, and the products were quantified on Microplate reader (BioTek, FLx800). Barcodes were ligated to the forward primer to distinguish between the samples (Szulejko et al., 2014). Based on the equal mass principle, the volume required for each sample was calculated and the PCR products were thoroughly mixed. Finally, the PCR amplification products were purified and sequenced on the Illumina Nova6000 platform (Guangzhou, China) (Rognes et al., 2016). Taking 1 μl library and do 2100 quality inspection with Agilent High Sensitivity DNA Kit on Agilent Bioanalyzer machine. The microbiome biological information was analyzed using QIIME2 (version 2019.4) (Martin, 2011). The sequences are available in NCBI Sequence Read Archive (SRA) database with the accession number SRP509138.

### Statistical analysis

Statistical analysis and graphics were processed using R x64 4.1.3 and Origin2021. In order to obtain the species classification corresponding to each OTU (operational taxonomic unit), the RDP classifier Bayesian algorithm was used to perform taxonomic analysis on 100% similar levels of OTU sequences, and the bacterial 16S rRNA database was Silva, and the results were displayed in the form of heatmaps. The Shannon index was measured in R using the vegan package. To evaluate the impact of different generations and treatments on microbial communities, principal coordinate analysis of the weighted UniFrac distance metric was performed using the ape package. Significance among different treatments was tested by multivariate analysis of variance using Origin. Random forest model classification was measured in R using the randomForest and rfPermute packages. In the first step, a random forest is used to identify important OTUs. Low-abundance OTUs that contributed poorly to classification and affected computational efficiency, excluding values with total abundance below 5 was filtered to generate 500 decision trees. Then, the cross-validation auxiliary evaluation selected a specific number of OTUs, performs 10 times of 10-fold cross-repeated verification. According to the results, a certain number of OTUs was retained, and the random forest was recalculated, 35 OTUs and used for the classification random forest operation, and the significance *P*-value was performed using MeanDecreaseAccuracy, for the OTU mark blank of *P* ≥ 0.05, for the OTU mark “^*^” of 0.01 ≤ *P* < 0.05, for the OTU mark “^**^” of 0.001 ≤ *P* < 0.01, For the OTU mark “^***^” of *P* < 0.001, the bar chart is finally drawn with the first 35 OTUs.

## Results and discussion

### The degradation rate of phenanthrene during the domestication process

**Figure 1:**
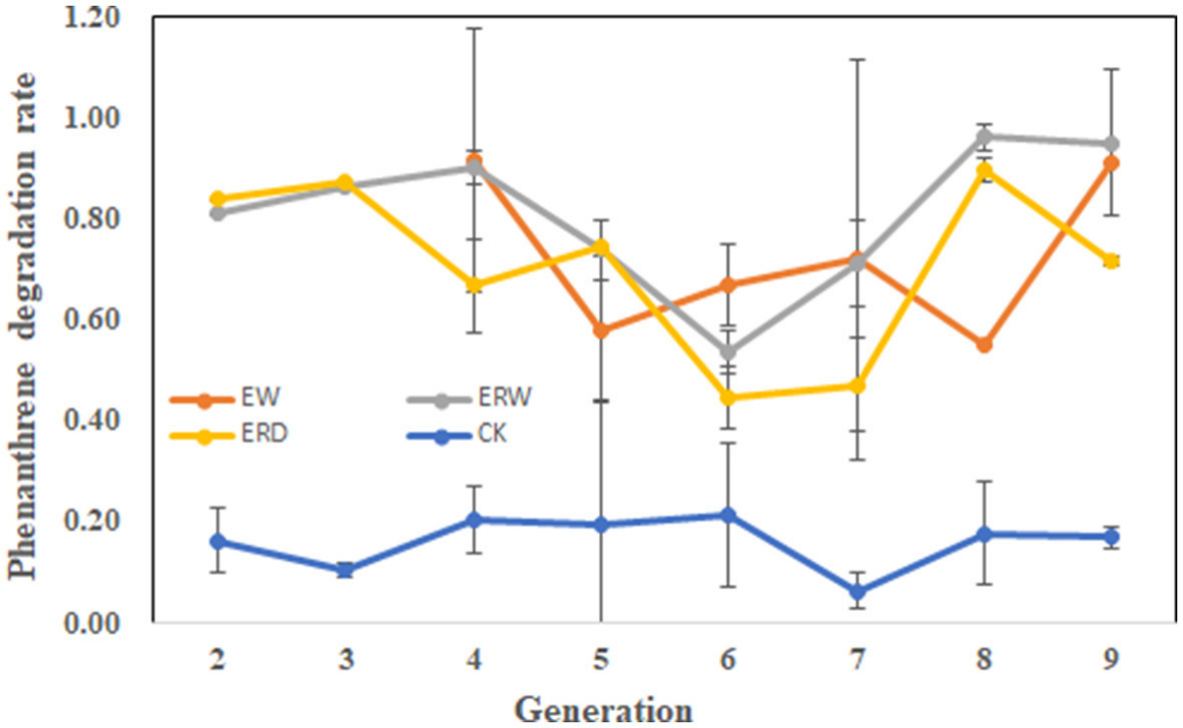
The phenanthrene degradation rate of each microbial generation under three different treatments of contaminated sludge containing PHE (EW), contaminated sludge containing PHE and pyrene (ERW) and tank truck sludge containing PHE and pyrene (ERD).

Figure 1 shows the PHE degradation rate of each generation of microbial communities for phenanthrene pollutants under three different treatments of contaminated sludge containing PHE (EW), contaminated sludge containing PHE and pyrene (ERW) and tank truck sludge containing PHE and pyrene (ERD). On the whole, the tendency of degradation rate was constantly fluctuated in each generation of all the treatments. The degradation rate of each generation exceeds 50%, indicating that each microbial generation had strong ability to degrade phenanthrene. It can be seen that the PHE degradation rate at first two generations was exceed to 80% in both contaminated sludge (ERW) and tank truck sludge (ERD), and then gradually decreased in generations from 4 to 7, but then dramatically increased in last two transferring processes. In the end, the final PHE degradation ratio of each treatment at the nine generation was ranked ERW > EW > ERD. The fluctuation of degradation ratio in each generation probably related to the difference of microbial community structure in each generation, contributing an unstable degradation ratio in each generation. At first two generations, the residual soil particles could provide protection to microbial communities and adsorb PHE to their surface, which accelerating the PHE biodegradation ratio. While after several transfer processes, soil particles were removed and microbes were slowly adapted and domesticated through interacting with PHE directly. Finally, microbes that could resistant and/or degrade PHE have survival with a stable and high PHE degradation ratio (Figure 1). Environmental alteration is a selection strategy that can lead to the loss of groups of organisms that were sensitive to the changed environmental factors, with cascading effects on ecosystem function (Carlisle and Clements, 2005). The change of degradation rate had a great relationship with the internal composition of bacteria, so further analysis of species abundance and diversity was needed.

### The microbial composition at the genus level during the domestication process

**Figure 2:**
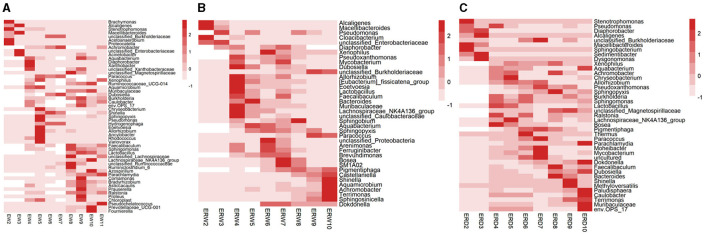
The relative abundance of bacteria at the genus level in the three treatments of contaminated sludge containing PHE (EW) **(A)**, contaminated sludge containing PHE and pyrene (ERW) **(B)** and tank truck sludge containing PHE and pyrene (ERD) **(C)**. The number of OTUs is represented from dark to light according to color. The symbols “*” and “**” represent *P* < 0.05 and *P* < 0.01, respectively.

By comparison, the enriched microbes collected from contaminated sludge containing phenanthrene (EW) changed from *Stenotrophomonas, Acetoanaerobium, Proteocatella, Alcaligenes*, Macellibacteroides, *Acinetobacter* to *Chryseobacterium, Sphingopyxis, Pseudomonas, Allorhizobium, Ancylobacter, Rhodococus, Shinella, Achromobacter*, and finally *Comamonas, Prauserella, Proteus, Lachnospiraceae_NK4A136_*group, *Azospirillum*, and *Pseudochelatococcus* dominated in the nine generation (Figure 2A). While the enriched microbes collected from the co-cultivation of phenanthrene and pyrene in contaminated sludge (ERW) changed from *Alcaligenes, Macellibacteroides, Cloacibacterium, Pseudomonas, Allorhizobium, Lactobacillus, Lachnospiraceae_NK4A136_group, Sphingobium* to *Bacteroides, Aquabacterium, Xenophilus, Bosea, SM1A02*; In the nine generation, the microbes mainly composed of *Sphingobium*, unclassified_*Burkholderiaceae, Paracoccus, Shinella, Aquamicrobium, Achromobacter, Terrimonas* and *Sphingosinicella* (Figure 2B). Additionally, under the co-cultivation of phenanthrene and pyrene (ERD), the microbes collected from the contaminated tank truck sediment changed from *Stenotrophomonas, Sphingobacterium*, Macellibacteroides to *Bosea, Lachnospiraceae_NK4A136*_group, unclassified_*Burkholderiaceae, Paracoccus, Moheibacter, Mycobacterium, Achromobacter* and *Sphingopyxis*, eventually stabilized to unclassified_*Magnetospirillaceae, Shinella* and *Methyloversatilis* (Figure 2C). These data showed that the composition of functional microorganisms changed during the passaged process under the same treatment, which mainly indicated that the functional redundancy and stability of microbial communities to keep a high biodegradation ratio of PHE (Figure 1). Comparing the three groups of EW, ERW, and ERD, it could be seen that the functional microorganisms between different treatments were also different, even their PHE degradation potential was strong, implying that the PHE degrading microbial community structure were various.

Among them, *Achromobacter* appeared under all three different treatments. It was previously reported that *Achromobacter* have the ability to degrade hydrocarbons (Deng et al., 2014). For example, *Achromobacter* isolated from polluted desert soil and petrochemically polluted environment have the ability to degrade phenanthrene (Ronen et al., 2000; Janbandhu and Fulekar, 2011). *Pseudomonas* appeared in both treatments of sludge from polluted water. Similarly, *Pseudomonas* are also commonly known in the biodegradation of PHE (Lei et al., 2002; Zavala-Meneses et al., 2024). However, even though *Achromobacter* appeared in the abundant species between different treatments, their appearance time was very different, and some bacteria were lost during the passage process, so we guessed that these reasons also led to a fluctuating change of phenanthrene degradation rate change detected.

### The variation of microbial communities during the domestication process

**Figure 3:**
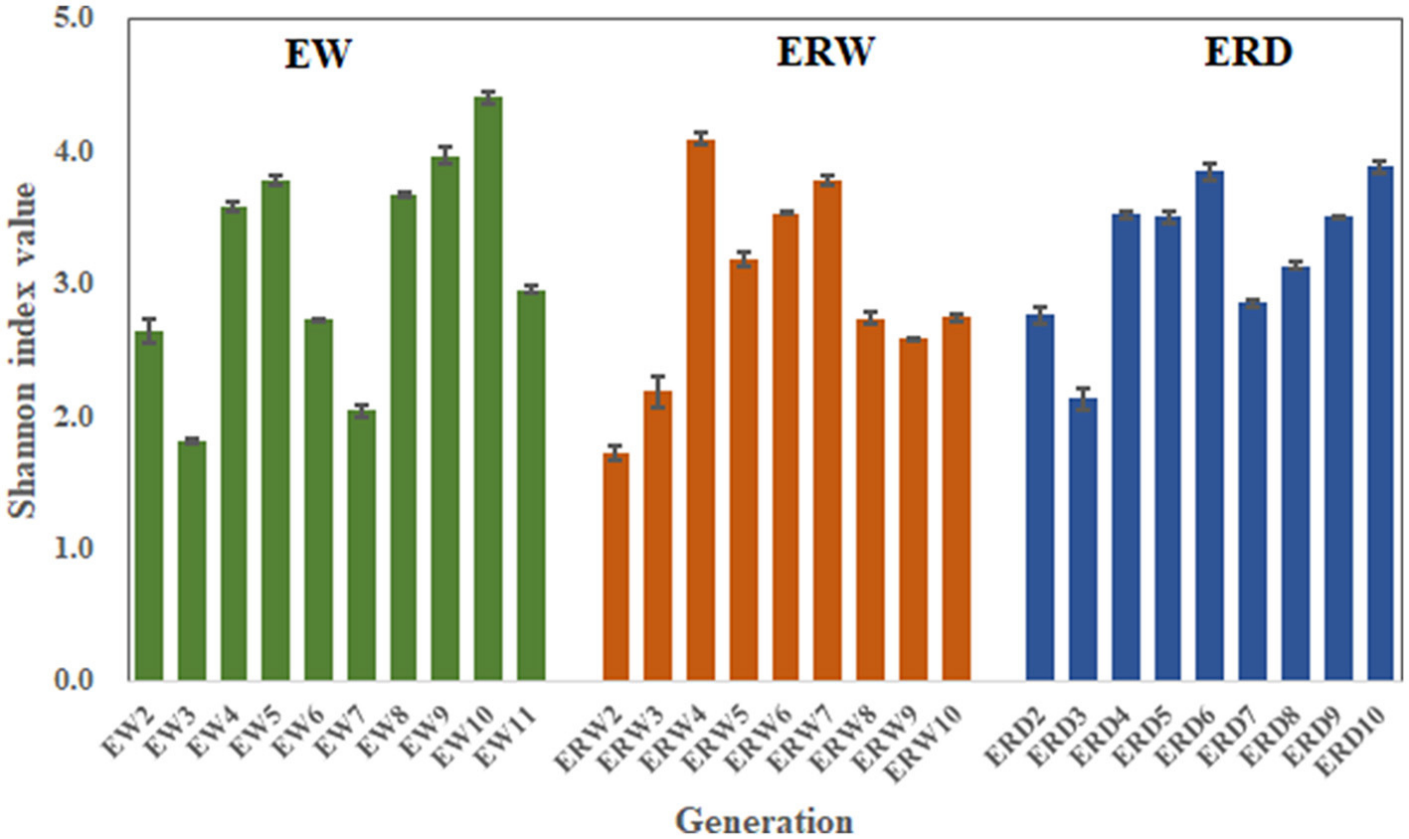
Shannon index of PHE-degrading bacteria under three treatments of contaminated sludge containing PHE (EW), contaminated sludge containing PHE and pyrene (ERW) and tank truck sludge containing PHE and pyrene (ERD).

**Figure 4:**
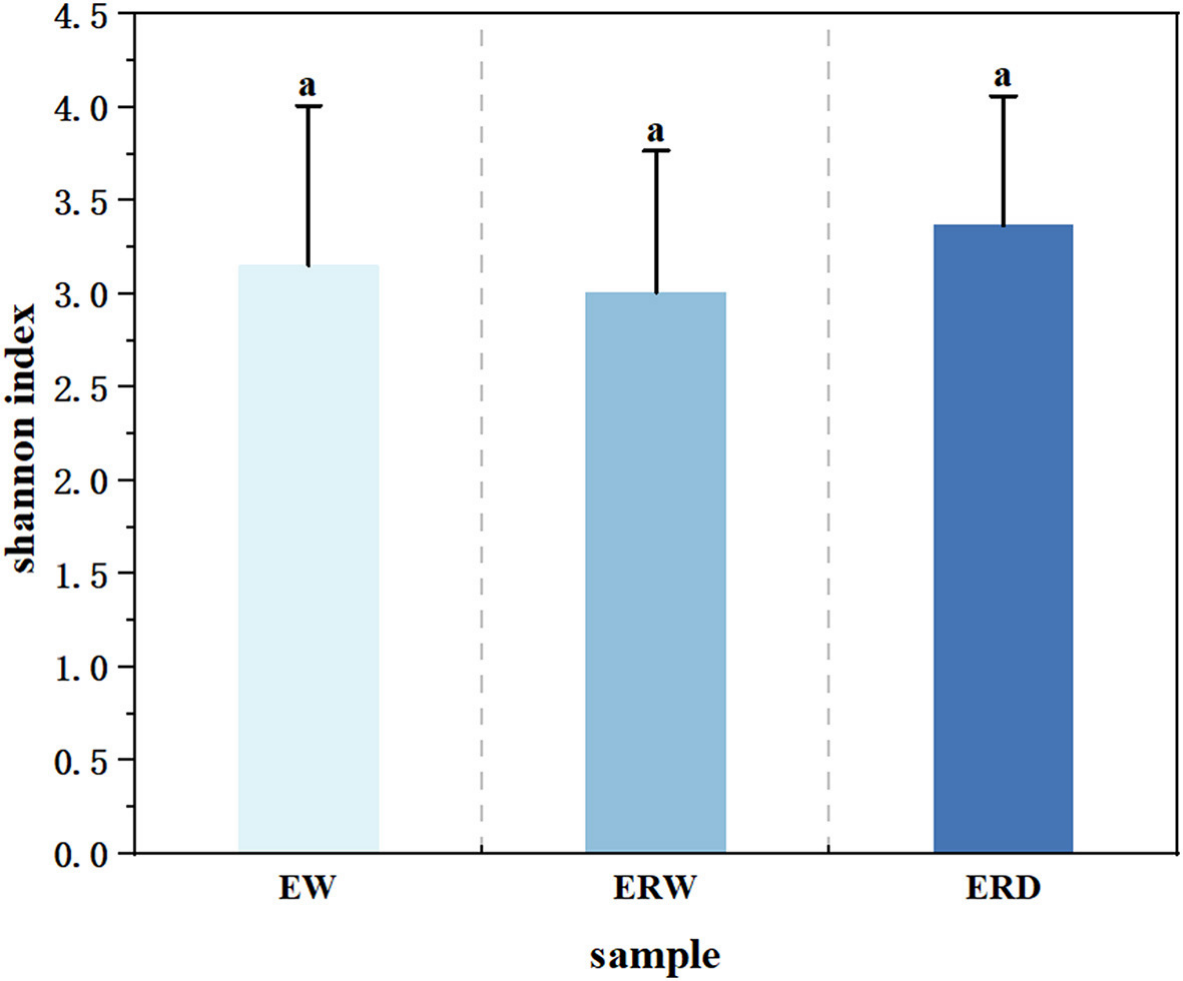
The average Shannon index of nine generation among three treatments of contaminated sludge containing PHE (EW), contaminated sludge containing PHE and pyrene (ERW) and tank truck sludge containing PHE and pyrene (ERD). ^a^Different lowercase letters represent significant differences.

In order to analyze the richness of the functional flora of phenanthrene degradation under three different cultures, and to better understand the differences of community structure, we performed α-diversity analysis to calculate the Shannon index (Figures 3, 4). The Shannon index is a classic index that reflects the diversity of ecosystem species by combining species richness and species abundance evenness (Lemos et al., 2011). The Shannon index of EW, ERW and ERD was ranged from 1.81 to 4.41, from 1.72 to 4.10 and from 2.11 to 4.61, respectively (Figure 3). The Shannon value of ERD was relatively high, indicating that the phenanthrene-degrading bacteria had a high diversity tank truck sludge. After nine transferring processes, the microbial community diversity ranking of EW > ERD > ERW. But there was no significant difference of microbial community diversity between these three treatments whining summarizing all nine transferring processes (Figure 4). Compared with the microbial composition of the tank truck bottom sludge, the Shannon index of the contaminated sedimentation group was relatively low.

**Figure 5:**
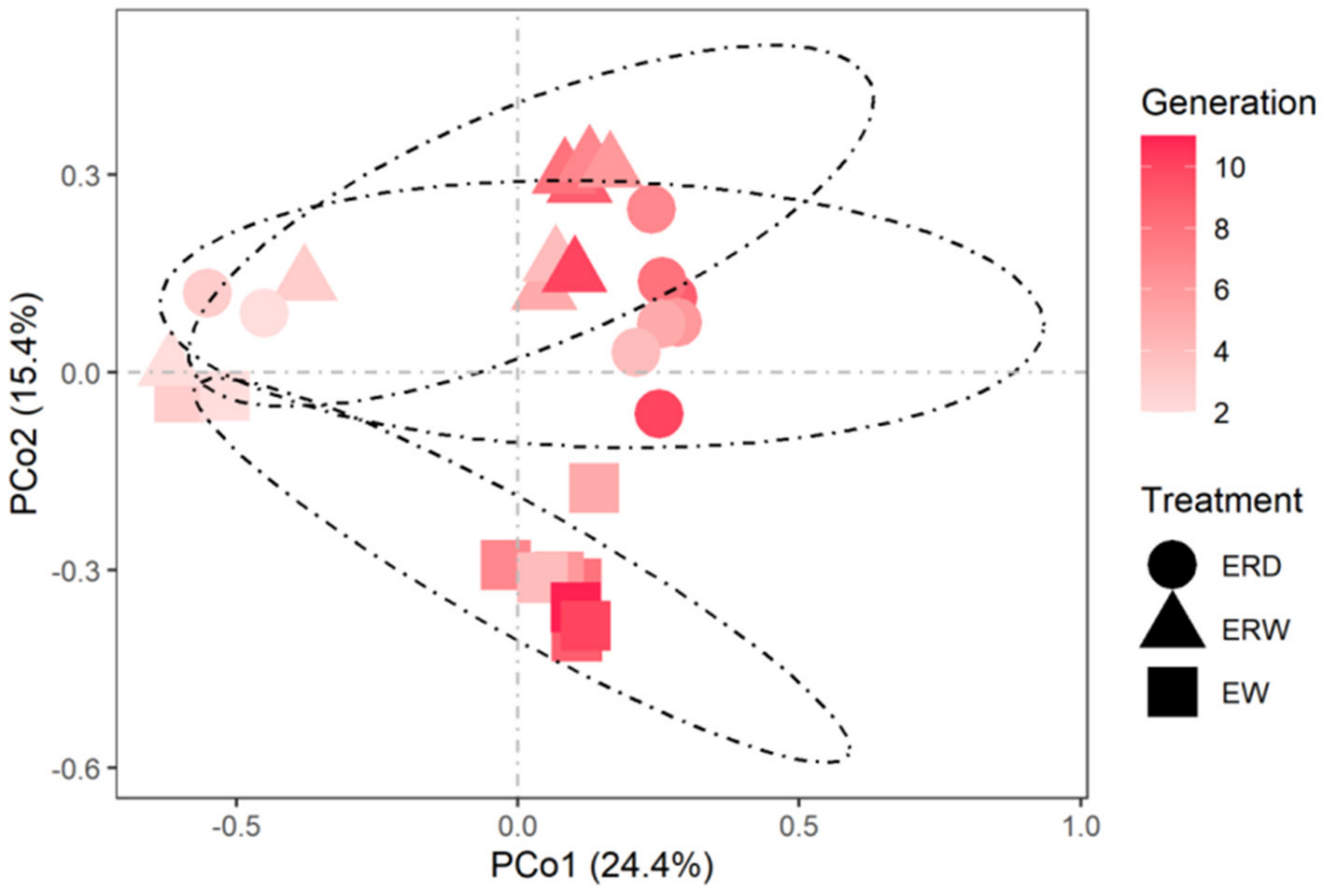
PCoA reflected the differences and distances in the composition of microbial communities under three different treatments of contaminated sludge containing PHE (EW), contaminated sludge containing PHE and pyrene (ERW) and tank truck sludge containing PHE and pyrene (ERD).

Principal component analysis (PCoA) was performed on the microbial communities under different treatments to reflect the differences in the composition of phenanthrene-degrading bacteria under three different treatments (Figure 5). The contribution rates of the first principal component and the second principal component were 24.4 and 15.4% respectively, and the cumulative contribution ratio reached to 39.8%. It is concluded that there were obvious distribution differences in the composition of microbial community under the three treatments. PERMANOVA results and pairwise analysis also showed that microbial communities enriched from EW vs. ERD and EW vs. ERW varied significantly (Table 1; *P* < 0.01). In these three samples, bacterial communities were significantly different along the *X*-axis at different culture times, and microorganisms were differentiated along the *Y*-axis. The results showed that time was the main factor causing differences in the microbial communities of the samples, followed by different treatments. Under different treatments, ERW had a similar bacterial community structure to ERD (*P* > 0.05), while EW had a large change in bacterial community composition, similar result can be found in pairwise analysis (Table 1).

**Table 1:** PERMANOVA results using Bray-Curtis distance metrics for the microbial communities and pairwise analysis between different samples.

**Variables**	**Df**	**Sums Of Sqs**	**Mean Sqs**	**F.Model**	***R* ^2^**	***P*-value**
Generation	9	4.04	0	0	0.43	>0.05
Sludge	2	1.67	1	0	0.18	>0.05
Generation: Sludge	16	3.59	0	0	0.39	>0.05
Res	0	0			0	
Total	27	9.30			1	
**Pairwise analysis**			
**Sludge**	**F.Model**	*R* ^2^	***P*** **-value**
EW vs. ERW	3.56	0.17	**0.003**
EW vs. ERD	3.02	0.15	**0.006**
ERW vs. ERD	2.13	0.12	>0.05

Bold values represent significant differences at *P* < 0.05.

The abundance, composition, and diversity of microbial communities were strongly related to changes in environmental gradients (Bier et al., 2015; Fan et al., 2016). There was not much difference in the similarity of the initial community of two different samples, but with the change of cultured environment, the difference of community composition gradually become larger. Even though the polluted sludge and oil tank truck sludge were two different samples, the final community composition was relatively close since they were both cultivated under the phenanthrene-pyrene pollutant. Therefore, we believed that the step-by-step screening of environmental gradients driven the aggregation of taxa with similar community structure and function, which was also consistent with the research results of Crump et al. (2012).

### The importance ranking of microbial groups during biodegradation of PHE

**Figure 6:**
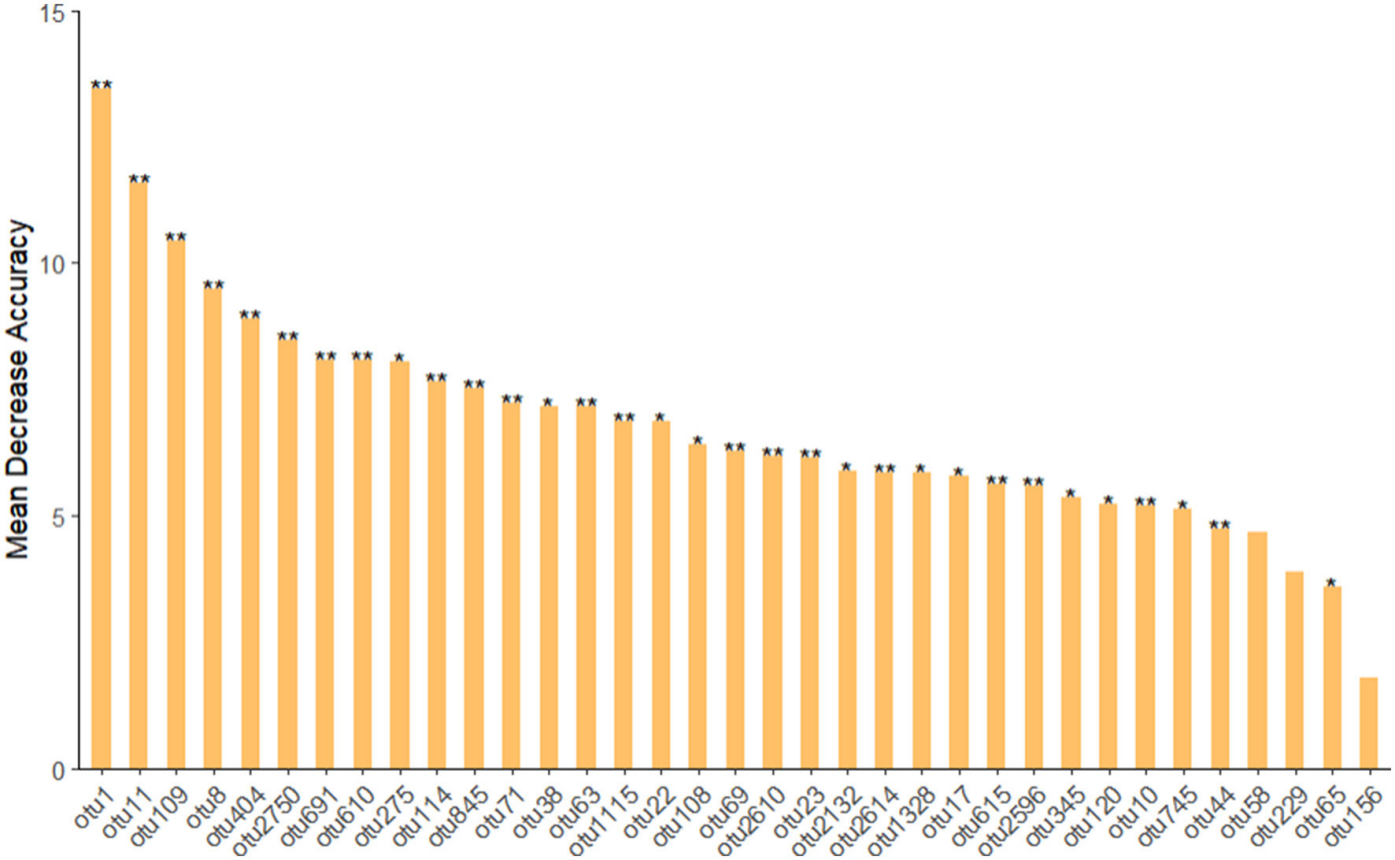
The importance of microbes using random forest model classification.

The calculation was based on phenanthrene-contaminated sedimentation sludge samples (ERW). We can find that the main OTUs were mainly affiliated to *Achromobacter, Pseudochelatococcus, Pseudoxanthomonas, Pseudomonas, Ochrobactrum, Kaistia*, Chloroplast, *Exiguobacterium, Ochrobactrum*, Muribaculaceae (Figure 6 and Table 2). Among them, *Achromobacter* has the most significant impact on the composition of the phenanthrene degrading group community, and *Achromobacter* appeared in the microbial composition of the previous three cultures. In addition, *Pseudoxanthomonas, Ochrobactrum* also have the function of degrading phenanthrene (Patel et al., 2012). This was basically consistent with the previous phenanthrene-degrading bacterial community composition. A series of diversity analyses were carried out on the obtained species, and the final results were that in the phenanthrene degrading bacterial community, the microbial composition was different between different generations, and the microbial composition between different treatments was also different, which mainly related to the result of adapting to the diversity of the environment and functional redundancy of microbial communities. Therefore, when we studying and enriching the functional degrading microbes of phenanthrene or other pollutants, more concern should be paid in not only the degradation rate, but also the diversity variation of microbial community composition.

**Table 2:** The detailed classification information of microbes in Figure 6.

**SampleID**	**Phylum**	**Class**	**Family**	**Genus**
OTU1	Proteobacteria	Betaproteobacteria	Burkholderiaceae	*Achromobacter*
OTU11	Proteobacteria	Alphaproteobacteria	Beijerinckiaceae	*Pseudochelatococcus*
OTU109	Proteobacteria	Gammaproteobacteria	Xanthomonadaceae	*Pseudoxanthomonas*
OTU8	Proteobacteria	Gammaproteobacteria	Pseudomonadaceae	*Pseudomonas*
OTU404	Proteobacteria	Alphaproteobacteria	Rhizobiaceae	*Ochrobactrum*
OTU2750	Proteobacteria	Betaproteobacteria	Burkholderiaceae	*Hydrogenophaga*
OTU691	Cyanobacteria	Oxyphotobacteria	Chloroplast	*Chloroplast*
OTU610	Firmicutes	Bacilli	Family_XII	*Exiguobacterium*
OTU275	Proteobacteria	Alphaproteobacteria	Rhizobiaceae	*Ochrobactrum*
OTU114	Proteobacteria	Alphaproteobacteria	Beijerinckiaceae	*Bosea*
OTU845	Bacteroidetes _	Bacteroidia	Chitinophagaceae	*Terrimonas*
OTU71	Proteobacteria	Gammaproteobacteria	Xanthomonadaceae	*Pseudoxanthomonas*
OTU38	Chlamydiae	Chlamydiae	Parachlamydiaceae	*Parachlamydia*
OTU63	Proteobacteria	Betaproteobacteria	Burkholderiaceae	Unclassified
OTU1115	Proteobacteria	Alphaproteobacteria	Caulobacteraceae	*Phenylobacterium*
OTU22	Proteobacteria	Gammaproteobacteria	Pseudomonadaceae	*Pseudomonas*
OTU108	Bacteroidetes	Bacteroidia	Chitinophagaceae	*Terrimonas*
OTU69	Proteobacteria	Gammaproteobacteria	Xanthomonadaceae	*Pseudoxanthomonas*
OTU2610	Proteobacteria	Betaproteobacteri	Rhodocyclaceae	Unclassified_Rhodocyclaceae
OTU23	Proteobacteria	Alphaproteobacteria	Azospirillaceae	*Azospirillum*
OTU2132	Planctomycetes	Planctomycetacia	Gimesiacea	Uncultured
OTU2614	Unclassified	Unclassified	Unclassified	Unclassified
OTU1328	Proteobacteria	Betaproteobacteria	Burkholderiaceae	*Pusillimonas*
OTU17	Firmicutes	Bacilli	Lactobacillaceae	*Lactobacillus*
OTU615	Proteobacteria	Gammaproteobacteria	Xanthomonadaceae	Luteimonas
OTU2596	Proteobacteria	Alphaproteobacteria	Rhizobiaceae	*Aquamicrobium*
OTU345	Proteobacteria	Betaproteobacteria	Burkholderiaceae	Unclassified
OTU120	Proteobacteria	Alphaproteobacteria	Rhodobacteraceae	*Paracoccus*
OTU10	Firmicutes	Clostridia	Lachnospiraceae	Lachnospiraceae_NK4A136_group
OTU745	Proteobacteria	Alphaproteobacteria	Midichloriaceae	Candidatus_Jidaibacter
OTU44	Bacteroidetes	Bacteroidia	Prevotellaceae	Prevotellaceae_UCG-001
OTU58	Firmicutes	Clostridia	Lachnospiraceae	Lachnospiraceae_NK4A136_group
OTU229	Proteobacteria	Alphaproteobacteria	Xanthobacteraceae	Uncultured
OTU65	Planctomycetes	Planctomycetacia	Isosphaeraceae	*Paludisphaera*
OTU156	Bacteroidetes	Bacteroidia	Muribaculaceae	*Muribaculaceae*

## Conclusion

Through the screening of phenanthrene-degrading bacteria under different culture conditions of contaminated sludge and tanker sludge, the tendency of degradation rate was constantly fluctuated in each generation of all the treatments. And the degradation rate of each generation exceeds 50%, indicating that each microbial generation had strong ability to degrade phenanthrene. Finally, we obtained a series of bacteria with phenanthrene-degrading functions such as *Achromobacter, Pseudomonas, Pseudochelatococcus, Bosea*, etc. Microbial community structure enriched during the phenanthrene degrading process was dramatically different among contaminated sludge and tanker sludge, which was mainly caused by the difference of environmental properties. Moreover, the community composition of phenanthrene-degrading bacteria did not tend to be stable with the passage processes since that the composition of the bacterial community was constantly changed and a variety of microorganisms participated in degrading phenanthrene pollutants during the domestication process, which implying that functional redundancy of microbial community was beneficial to keep a powerful degrading potential of phenanthrene. Additionally, the functional microbes can be enriched and applied to the *in-situ* soil remediation in further study.

## Data availability statement

The original contributions presented in the study are publicly available. This data can be found here: NCBI SRA, accession SRP509138.

## Author contributions

GZ: Writing – review & editing, Writing – original draft, Methodology, Formal analysis. HQ: Writing – original draft, Methodology, Investigation, Data curation. YL: Writing – review & editing, Resources, Methodology, Investigation, Formal analysis. XY: Writing – review & editing, Visualization, Validation, Resources, Project administration, Funding acquisition. XN: Writing – original draft, Resources, Investigation.
